# Bedside EEG for rapid diagnosis of delirium

**DOI:** 10.1016/j.neurot.2025.e00768

**Published:** 2025-10-21

**Authors:** Takehiko Yamanashi, Tsuyoshi Nishiguchi, Gen Shinozaki

**Affiliations:** a Department of Neuropsychiatry, Faculty of Medicine, Tottori University, Yonago, Tottori, Japan; b Department of Psychiatry and Behavioral Sciences, Stanford University School of Medicine, Palo Alto, CA, USA

**Keywords:** Delirium, Electroencephalogram, EEG, Bispectral EEG, BSEEG

## Abstract

Delirium is a frequent complication among older adults and is linked to higher mortality, longer hospital stays, and greater healthcare expenditure. Although its clinical relevance is well recognized, routine diagnosis remains challenging because existing tools rely largely on the observation of fluctuating symptoms, which can easily be overlooked in daily practice. Electroencephalography (EEG) provides an objective measure of brain activity, and characteristic changes such as generalized slowing have been consistently described in patients with delirium. Despite these established findings, the conventional EEG setup is technically demanding and not well suited for repeated use in general hospital wards. In recent years, portable point-of-care (POC) EEG systems have been developed, allowing recordings with a limited number of electrodes at the bedside. Several clinical studies have reported that these devices are able to detect delirium with acceptable accuracy, while also offering practical advantages such as rapid deployment and use by non-specialist staff. Among the approaches investigated, the bispectral EEG (BSEEG) method has attracted particular attention. A higher BSEEG score has been shown to correlate with delirium severity and to predict adverse outcomes, including reduced survival, even in patients who did not present with overt clinical symptoms. Beyond the clinical setting, experimental work has applied EEG and BSEEG to rodent models of delirium induced by inflammation or surgery. These studies have highlighted associations between EEG slowing, microglial activation, and behavioral disturbances, suggesting that electrophysiological changes may provide a translational link between basic mechanisms and clinical phenomena. Preclinical data also indicate that BSEEG could serve as a quantitative tool for assessing treatment response in experimental models. Taken together, these findings support the potential of simplified EEG platforms to complement current diagnostic strategies. If further validated in larger, real-world cohorts, bedside EEG may become a practical adjunct for the early recognition of delirium and the monitoring of disease progression, with implications for both patient outcomes and mechanistic research.

## Introduction

In the context of an aging society, delirium—where age is the most significant risk factor—is an increasingly critical medical challenge. Delirium is a confusional state with acute onset associated with poor prognosis, particularly among elderly hospitalized patients.

For instance, in the United States, approximately 12 million elderly individuals (aged 65 and older) are hospitalized annually. Reports indicate that up to 50 ​% of patients in general medical wards or post-surgical settings, and nearly 90 ​% of patients in intensive care units, develop delirium [1,2]. This translates to an estimated 2 to 3 million cases of delirium each year. Patients with delirium face heightened risks of complications due to impaired attention and confusion, often leading to longer hospital stays compared to those without delirium [3,4]. Furthermore, the mortality rate of patients with delirium is higher, and the associated costs—including discharges to long-term care facilities due to complications—are estimated at roughly $60,000 per case [[5], [6], [7]]. The occurrence of delirium presents various challenges; Therefore, early diagnosis and appropriate intervention are highly desirable.

However, no objective and effective diagnostic methods have been firmly established to date, often leading to delays in appropriate interventions [8]. Over the past three decades, efforts have been made to facilitate early detection of delirium. These methods, however, largely rely on questionnaire-style screening tools conducted by frontline hospital staff and have yet to see widespread adoption in busy clinical settings.

## Detection of Delirium

In clinical practice, the detection and diagnosis of delirium are performed by assessing the manifested symptoms and behaviors. The diagnostic criteria in the DSM-5-TR are the “gold standard”, describing the clinical presentation as “disturbance in attention and awareness” accompanied by “an additional disturbance in cognition” [9]. Determining whether these symptoms are present can be particularly challenging, especially when symptoms are mild. Therefore, although the DSM-5-TR diagnostic criteria, which also refer to symptom fluctuations and medical causes, are essential, they are not practical for many medical staff in routine practice. To facilitate broader use, various screening tools have been developed to efficiently detect delirium symptoms [[10], [11], [12]]. The Confusion Assessment Method (CAM), was one of the earliest screening tools [13], with derivatives such as the CAM-ICU, designed for non-verbal patients in intensive care units [14]. Additionally, faster assessment tools include the 3D-CAM, which can be completed in about 40 ​s [15], and the ultra-brief CAM (UB-CAM), which incorporates the two-item Ultra-Brief screener (UB-2) paired with the 3D-CAM [16]. Other simple screening tools include the 4 'A's Test (4AT) [17] and the Nursing Delirium Screening Scale (Nu-DESC) [18]. The Delirium Observation Screening Scale (DOSS) relies solely on nurses' observations [19]. The Single Question in Delirium (SQiD) is the simplest screening tool, which attempts to detect changes using a single query [20]. There are also more detailed evaluation tools for capturing delirium symptoms, including the Delirium Rating Scale-Revised-98 (DRS-R-98) [21] and the Memorial Delirium Assessment Scale (MDAS) [22]. While all these tools assess clinically observable symptoms, their evaluations may vary depending on the individual hospital staff performing the assessment and are prone to subjectivity. Furthermore, their implementation often requires considerable time and effort, presenting practical limitations. To overcome these limitations, objective methods for detecting delirium need to be widely adopted.

## Delirium and EEG

Electroencephalography (EEG) has been proposed as a valuable tool for the detection of delirium. EEG involves the placement of conductive electrodes on the scalp to record electrical activity in the brain. Using amplifiers and voltmeters, these recordings capture instantaneous voltage changes, providing a time-resolved depiction of neural activity. Clinically, EEG studies typically use 16 to 21 electrodes, although advanced high-density systems can record from 128 or more scalp locations simultaneously. Although EEG has a lower spatial resolution, it has a higher time resolution compared to neuroimaging techniques such as MRI or PET, enabling the investigation of brain activity fluctuations in vivo on timescales matching cognitive processes. Furthermore, EEG can be performed at the bedside in acutely ill or postoperative patients, making it a more practical modality for studying delirium compared to other imaging tools such as MRI, fMRI, or PET [23].

In awake, healthy adults, EEG patterns generally show a combination of low-amplitude, high-frequency (13–25 ​Hz, termed beta) activity in frontal regions and moderate-frequency (8–13 ​Hz, termed alpha) activity over posterior regions. In contrast, EEG recordings from patients with delirium exhibit distinct alterations. In the 1940s and 1950s, Romano and Engel performed a series of EEG recordings in patients experiencing acute delirium due to cardiac or pulmonary disease or toxic ingestions. They noted that as cognitive function deteriorated, EEG traces displayed generalized slowing (increased delta activity), which subsequently normalized with treatments such as oxygen to restore cognitive function [24,25]. Based on these findings, the authors proposed that delirium represents a syndrome of cerebral insufficiency, wherein the brain's metabolic supply is insufficient to sustain normal function [26]. Since the early investigations, numerous researchers have reproduced similar findings [27,28]. In many studies, slow wave activity, characterized by high levels of delta and theta waves or low levels of alpha waves, has been demonstrated in subjects exhibiting delirium [[29], [30], [31], [32], [33], [34], [35], [36], [37], [38], [39], [40], [41], [42], [43], [44]].

In several previous studies, associations have been identified between abnormal EEG findings and the severity of delirium, as well as between EEG findings and clinical outcomes. Tanabe et al. demonstrated that slow-wave activity correlated with DRS, particularly within frequencies below 6 ​Hz, indicating that an increase in slow-wave activity was associated with greater delirium severity [39]. Guay et al. also showed that the severity of delirium symptoms was inversely correlated with occipital alpha relative power in eyes-closed states and positively correlated with occipital theta relative power in eyes-open states [45]. Kimchi et al. showed that delirium severity, as categorized by 3D-CAM-S scores, was strongly associated with the prevalence of generalized EEG slowing [46]. Surprisingly, it was the presence of generalized slowing of EEG activity, rather than the presence or absence of clinical symptoms of delirium, that was associated with outcomes such as mortality [46]. They further developed the EEG Confusion Assessment Method Severity (E-CAM-S) from routinely acquired EEG data using four frontal EEG channels based on a learning-to-rank machine learning model [47], and the Visual EEG Confusion Assessment Method Severity (VE-CAM-S) which consists of visually coded EEG features including generalized/diffuse delta and theta slowing, generalized rhythmic delta activity, loss of reactivity, and triphasic waves [48]. They demonstrated that both E-CAM and VE-CAM were associated with CAM-S, which reflects the clinical symptoms of delirium, as well as with outcomes including mortality [47,48]. These data clearly show that limited channel EEG can provide signals to capture delirium. Notably, Sloter et al. have systematically proven that. The characteristic EEG findings of delirium are often reported as “diffuse slowing”, indicating slow (delta or theta) wave observed diffusely across all channels. Thus, instead of relying on all of those multiple channels, it is likely that limited channels, potentially even one channel, can be used to detect delirium. They evaluated all combinations of two electrodes across multiple traditional EEG electrode settings, and demonstrated that single channel from two electrodes combination can show excellent performance [32].

## Delirium detection by portable EEG device or Point-of-Care EEG

**Fig. 1:**
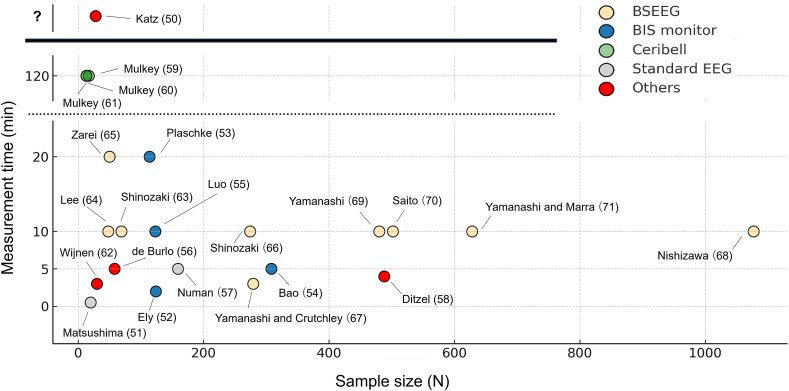
Scatter plot summarizing studies on delirium detection using portable or point-of-care EEG devices, showing measurement duration (vertical axis) and sample size (horizontal axis).

As the evidence mentioned above shows, EEG is considered useful for detecting delirium as it directly captures brain electrophysiological activity objectively. However, conventional EEG devices are typically large and require the expertise of experienced clinical technicians for precise electrode placement, making it challenging to perform routine and frequent examinations on a large number of patients. To address these limitations, efforts are being made to detect delirium using portable EEG devices, enabling Point-of-Care (POC) EEG assessments [49] (Table 1, Fig. 1).

**Table 1:** Summary of studies on delirium detection using portable or point-of-care EEG devices.

Ref	Authors	Publication year	Subjects	Total cases (delirium cases)	Recording time	Device	Electrodes	Region	Main findings in delirium subjects
[50]	Katz IR et al.	1991	Elderly residents of nursing home and congregate housing	28 (10)	unknown	Neurotrac (Interspec, Inc)	4	P3–O1, P2–O4	an increase in delta power, an increase in theta power, and a decrease in alpha power
[51]	Matsushima E et al.	1997	Acute myocardial infarction patients admitted to the CCU	20 (10)	30 ​s	Standard EEG	3	Fp1,C3, O1	reduced alpha wave activity and the appearance of low-amplitude theta waves
[52]	Ely EW et al.	2004	Mechanically ventilated adult ICU patients	124 (unknown)	2 ​min	BIS monitor	4	Frontal electrodes	no differences
[53]	Plaschke K et al.	2010	Post-op open-heart surgery patients	114 (32)	15–20 ​min	BIS monitor	4	Frontal electrodes	a eduction in relative alpha power and an increase in theta power
[63]	Shinozaki G et al.	2018	General hospital inpatients (elderly)	45 (4) ​+ ​24 (12)	10 ​min	BSEEG [CMS2100 (Contec)]	4	Fp1–A1, Fp2–A2	increased BSEEG score
[66]	Shinozaki G et al.	2019	Elderly inpatients; prospective BSEEG cohort	274 (102)	10 ​min	BSEEG [CMS2100 (Contec)]	4	Fp1–A1, Fp2–A2	relationships between high BSEEG score and higher mortality
[64]	Lee S et al.	2019	Emergency department patients	48 (9)	10 ​min	BSEEG [CMS2100 (Contec)]	4	Fp1–A1, Fp2–A2	increased BSEEG score
[57]	Numan T et al.	2019	Postoperative patients ≥60 years, undergoing planned surgery	159 (55)	5 ​min	Standard EEG	3	Fp2-Pz, T8-Pz	Increased Relative Delta Power (RDP, 1–4 ​Hz) after surgery in POD patients
[65]	Zarei K et al.	2020	Patients undergoing electroconvulsive therapy (ECT)	50 (N/A)	5–20 ​min	BSEEG [CMS2100 (Contec)]	4	Fp1–A1, Fp2–A2	increased BSEEG score after ECT
[70]	Saito T et al.	2021	dataset from Ref. [66] and newly recruited elderly patients (≥55)	502 (168)	5–10 ​min	BSEEG [CMS2100 (Contec)]	2	Fp1–A1	showed that the BSEEG score is a predictor of mortality in patients with dementia
[71]	Yamanashi T and Marra P et al.	2021	dataset from Refs. [63,66], and [70] and newly recruited patients	628 (N/A)	5–10 ​min	BSEEG [CMS2100 (Contec)]	2	Fp1–A1	showed that the BSEEG score is a predictor of mortality in patients with sepsis
[67]	Yamanashi T and Crutchley K et al.	2021	Elderly participants; new thumb-size BSEEG device	279 (93)	3 ​min	BSEEG [ZA (ProAssist)]	2	Fp1–A1	increased BSEEG score, correlation between BSEEG score and delirium severity
[69]	Yamanashi T et al.	2021	dataset from Ref. [66] and part of [67]	274 (102) ​+ ​206 (42)	3–10 ​min	BSEEG [CMAS2100 (Contec) or ZA (ProAssist)]	2	Fp1–A1	enhanced detection accuracy through topological data analysis
[55]	Luo A et al.	2021	Hospitalized psych-consult patients (non-neuro & dementia subgroups)	123 (40)	10 ​min	BIS monitor	4	Frontal electrodes	low theta/low delta ratio
[62]	Wijnen VJM et al.	2022	Patients ≥60 years with major neurocognitive disorder	30 (14)	3 ​min	MobiMini (Micromed)	4	Fp2-Pz, T8-Pz	increased Delta activity, decreased theta2 (6–8 ​Hz) activity, and decreased alpha activity on T8-Pz
[59]	Mulkey MA et al.	2022	Critically ill patients ≥50 years requiring mechanical ventilation >12 ​h	17 (9)	2 ​h	Ceribell device (Ceribell Inc.)	10	Covers all 5 cerebral lobes	higher theta/alpha ratios
[60]	Mulkey MA et al.	2022	dataset from [59]	13 (unknown)	2 ​h	Ceribell device (Ceribell Inc.)	10	Covers all 5 cerebral lobes	enhanced detection accuracy through machine learning methods.
[61]	Mulkey MA et al.	2023	dataset from [59]	13 (unknown)	2 ​h	Ceribell device (Ceribell Inc.)	10	Covers all 5 cerebral lobes	enhanced detection accuracy through a supervised deep learning Vision Transformer model
[68]	Nishizawa Y et al.	2023	dataset from Refs. [63,66,67], and [71]	1077 (334)	3–10 ​min	BSEEG [CMAS2100 (Contec) or ZA (ProAssist)]	2	Fp1–A1	dataset were normalized to combine, demonstrating that high BSEEG values were associated with increased mortality.
[54]	Bao L et al.	2023	Patients ≥75 years, elective non-cardiac, non-neuro surgery	308 (50)	5 ​min	BIS monitor	4	Frontal electrodes	lower BIS values
[58]	Ditzel FL et al.	2024	ICU patients ≥18 years or non-ICU patients ≥60 years	223 (94) ​+ ​265 (48)	4 ​min	DeltaScan (Prolira)	2	Fp2, Pz	an elevated score derived from polymorphic delta activity
[56]	de Burlo R et al.	2025	Inpatients >18 years assessed by psychiatry	58 (20)	5 ​min	SedLine (Masimo)	6	Frontal electrodes	higher HighDelta (2–4 ​Hz), lower LowAlpha(8–10 ​Hz)HighDelta (2–4 ​Hz) frequency band ratio

The practice of conveniently measuring EEG with limited leads at the bedside to evaluate abnormalities in brain function began in the 1990s [50,51]. Katz et al. conducted a prospective study on delirium and related encephalopathy in long-term care facilities. Using a two-channel EEG device, they repeatedly recorded brainwave data from parieto-occipital electrodes (P3–O1, P2–O4) and simultaneously assessed the Mini-Mental State Examination (MMSE) to capture the clinical symptoms of delirium. The study showed that patients with delirium exhibited an increase in delta power, an increase in theta power, and a decrease in alpha power [50]. Matsushima et al. employed a 16-channel electroencephalograph but recorded data exclusively from the central (C3) and occipital (O1) regions to investigate their relationship with delirium. They observed that reduced alpha wave activity and the appearance of low-amplitude theta waves were consistent with impaired attention, sleepiness, and confusion in delirious patients. Additionally, the theta/alpha ratio was significantly higher in the C3 and O1 regions in the delirious patient group, indicating a slow EEG activity were detectable with limited leads [51].

The bispectral index (BIS) monitor is an FDA-approved device that measures EEG data using four electrodes placed on the forehead. It displays BIS value to assess the depth of anesthesia, with lower BIS index values generally indicating deeper anesthesia; however, the algorithm used to calculate the BIS index has not been disclosed. Studies have been conducted to explore the use of the BIS for delirium detection. In the first investigation using BIS, mechanically ventilated adult patients in the intensive care unit (ICU) were evaluated to examine the relationship between the BIS value and the presence of delirium; however, the BIS value could not distinguish between patients with and without delirium [52]. In contrast, in a study targeting patients after cardiac surgery, the BIS value in those who developed delirium was significantly lower than in those who did not. Analysis of the raw BIS EEG data revealed a significant reduction in relative alpha power and an increase in theta power. Furthermore, a significant negative correlation was observed between plasma cortisol levels and the BIS index [53]. Another prospective study involving 308 elderly patients undergoing elective non-neurosurgical and non-cardiac procedures found that patients who developed POD had significantly lower BIS values both preoperatively and postoperatively compared with non-delirious patients. The ROC curve for the first postoperative BIS value showed that the AUC was 0.74 and the difference in BIS values was most pronounced on postoperative day 2 [54]. Luo et al. used BIS monitoring with density spectral array (DSA) to visualize the raw EEG amplitude in different frequencies. They found that low theta/low delta ratio was associated with delirium in non-neurological patients, and that low theta power was associated with delirium in patients with dementia [55]. The same research group recorded EEG data using another device SedLine and calculated the power of each EEG frequency band. They demonstrated that the HighDelta (2–4 ​Hz) band was positively associated with delirium diagnosis (AUC ​= ​0.87–0.88), while the LowAlpha (8–10 ​Hz)/HighDelta (2–4 ​Hz) frequency band ratio was inversely associated with delirium diagnosis (AUC ​= ​0.87–0.91) [56].

Numan et al. conducted a multicenter study using 159 surgical patients to develop a single-channel EEG approach to detect postoperative delirium. EEG recordings were performed preoperatively and during the first three postoperative days, alongside cognitive assessments. One-minute segments of raw EEG data were analyzed via spectral analysis to compute relative power metrics. They showed that Relative Delta Power (RDP, 1–4 ​Hz) after surgery detected delirium with an AUC of 0.75. The relative power from low-frequency power (1–6 ​Hz) on Fp2-Pz demonstrated the ability to detect delirium with an AUC of 0.78 (0.78, 95 ​% CI 0.72–0.84) than the relative delta power. Furthermore, RDP was significantly linked to delirium likelihood, severity, attentional deficits, and levels of consciousness [57]. Subsequently, they tested the delirium detection capability of the developed commercial device, DeltaScan [58]. EEGs were recorded for 4 ​min with DeltaScan, and an algorithm was applied to isolate the first 96 ​s free from artifacts for PDA detection. 494 data from ICU and non-ICU patients were included for data analysis. Although the analysis targeting delirium showed a modest AUC of 0.71, when they assessed acute encephalopathy, the AUC improved to 0.86 [58]. Although DeltaScan obtained FDA clearance for detection of acute encephalopathy in 2023, unfortunately Prolira, the company that produced DeltaScan, went bankrupt in 2024 and the current status or availability of the technology is unknown.

Mulkey et al. have evaluated the delirium detection capability of the 10-electrode headband EEG device created for seizure detection, known as Ceribell rr-EEG system [[59], [60], [61]]. In their first pilot study, a small cohort of 17 mechanically ventilated patients aged 50 years or older, including 9 with delirium, underwent 2-h EEG recordings using the Ceribell rr-EEG system. They reported that theta/alpha ratios were higher in patients with delirium [59]. Subsequently, using data from 13 out of 17 patients, they sought to enhance delirium detection accuracy through three machine learning methods: stepwise linear discriminant analysis (SWLDA), support vector machine (SVM), and random forest. They reported that SWLDA achieved the highest classification accuracy (97 ​%) in distinguishing delirium-positive and negative cases. SVM and random forest models yielded lower accuracies (74–82 ​% and ∼70 ​%, respectively). Gamma power ratios consistently differentiated cases, suggesting the potential of machine learning-enhanced EEG analysis as a delirium biomarker [60]. In addition, these datasets were analyzed with a supervised deep learning Vision Transformer (ViT) model. Preprocessed EEG data were transformed into image-like slices (e.g., 5-s segments) for classification using the ViT model, which achieved a testing accuracy of 97.58 ​% with optimally sized data slices. These findings underscore the significance of low-frequency signals, such as delta waves, in predicting delirium and highlight the promise of deep learning models for accurate detection [61]. However, given the very small sample size used for the machine learning analysis, which typically requires large datasets for reliable performance, caution is warranted in interpreting results based on fewer than twenty subjects until replication and validation with larger sample size is confirmed. As of August 2025, there is no follow up data reported based on this device.

The diagnosis of delirium superimposed on dementia (DSD) is sometimes difficult because of the symptomatic overlap between dementia and delirium. Wijnen et al. conducted a study involving 30 dementia patients, including 14 with DSD, to evaluate the MobiMini POC-EEG system using bipolar recordings from the right prefrontal and temporal regions. Post hoc EEG data processing with BrainVision Analyzer showed increased Delta activity, decreased theta2 (6–8 ​Hz) activity, and decreased alpha activity on T8-Pz in DSD. In particular, the analysis yielded a Delta/Theta2 (6–8 ​Hz) power ratio with an AUC of 0.80 for distinguishing DSD from dementia alone. However, since all patients received benzodiazepines or clozapine to manage severe agitation or psychotic symptoms, the generalizability of the findings requires further validation [62].

## BSEEG score and delirium

We have also worked on acquiring EEG data using compact EEG devices and developing algorithms for delirium detection. We first used a palm-size handheld device (CMS2100, Contec). Using this device, we obtained resting-state, eyes-closed EEG for approximately 10 ​min from two channels located bilaterally on the frontal region. To ensure the quality of the obtained data, we consulted a neurologist specializing in electrophysiology for accurate data acquisition and interpretation. We then performed spectral analysis to develop the most effective algorithm for distinguishing between delirium and non-delirium groups. Through iterative processes, we developed the “BSEEG (bispectral EEG) score” and verified its ability to detect delirium accurately. Participants in our first study were recruited from the general medicine and orthopedic departments of the University of Iowa Hospitals and Clinics. In the training dataset (45 patients, 184 recordings), the BSEEG index, with a cutoff of 1.44, achieved an accuracy of 87.5 ​%, a sensitivity of 80 ​%, and a specificity of 87.7 ​% (AUC ​= ​0.70). Validation in an additional group of 24 subjects yielded consistent results, with an accuracy, sensitivity, and specificity of 83.3 ​% for each (AUC ​= ​0.81) [63]. Among 48 patients in an emergency department (ED) setting, the BSEEG index, with a cutoff value of 1.45, demonstrated high diagnostic accuracy, achieving an AUC of 0.91, a sensitivity of 88.9 ​%, and a specificity of 92.3 ​% [64]. Furthermore, BSEEG scores were longitudinally obtained from 50 patients undergoing electroconvulsive therapy (ECT). The results demonstrated an increase in BSEEG values after ECT compared to baseline levels, with a gradual recovery period of 2–3 ​h required for the BSEEG scores to return to baseline. These findings suggest that BSEEG scores can objectively quantify patients' levels of confusion after ECT-induced seizures [65].

**Fig. 2:**
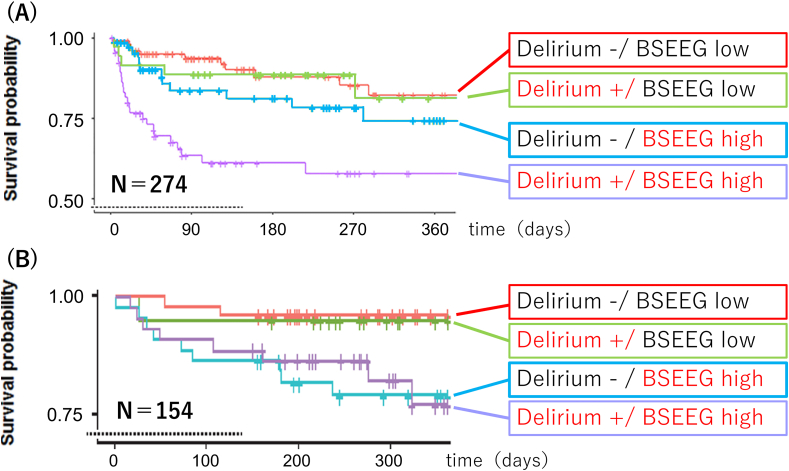
Kaplan–Meier cumulative survival curves over one year based on both clinical delirium status and BSEEG category. (A) Adapted from reference [66]. (B) Adapted from reference [67].

As shown in other studies [46,48], the importance of detecting delirium lies in its significant impact on patient outcomes, including survival. If BSEEG can accurately detect delirium, it might also predict prognosis. To test this hypothesis, the relationship between BSEEG scores and clinical outcomes was examined in 274 hospitalized patients. Patients were divided into high BSEEG score (indicating the presence of slow wave) and low BSEEG score groups based on measurements taken immediately after admission. The results showed that the one-year survival rate was significantly lower for the high BSEEG group. Furthermore, when stratifying BSEEG scores into low, medium, and high groups, a dose-dependent decrease in survival rates was observed with increasing BSEEG scores. Interestingly, a group with high BSEEG scores but no clinical symptom-based diagnosis exhibited the second-lowest survival rate, following the group clinically diagnosed with delirium and classified as high BSEEG group (Fig. 2-A). These results are strikingly consistent with those reported by Kimchi et al. based on conventional EEG data [46] and suggest that BSEEG may be able to identify high-risk groups that cannot be detected by current clinical practice based on symptom assessment [66].

To advance the practical application of BSEEG technology, it is essential to develop user-friendly devices for frontline medical staff. Using a more simplified thumb-size EEG device (ZA, Proassist Co., Ltd), the BSEEG algorithm was further evaluated. In this device, one channel EEG data was acquired with two electrodes; one location on the forehead and one location behind the ear. Measurements were conducted 612 times from 279 patients, and the results confirmed that higher BSEEG scores correlated with greater delirium severity as measured by the Delirium Rating Scale (DRS). Furthermore, outcomes predicted based on BSEEG scores using this new device were consistent with findings from the earlier device, demonstrating worse outcomes in groups with higher BSEEG scores independent of delirium clinical status (Fig. 2-B) [67].

Ultimately, data from 1,077 participants were integrated with a normalization of the BSEEG values across different devices to ensure consistency. This integrated dataset confirmed that the previous results could be reproduced regardless of age. Further subgroup analyses examined the ability of normalized BSEEG scores to predict long-term outcomes regardless of different delirium motor subtypes (hyperactive and hypoactive delirium). Significant predictive power was observed for hypoactive delirium, while similar trends were noted in hyperactive delirium despite the small sample size precluding statistical significance [68]. We further transformed the obtained EEG data using topological data analysis (TDA) and were able to improve the detection capability for delirium [69]. Moreover, we demonstrated that the BSEEG score is a predictor of mortality in patients with dementia or sepsis [70,71].

The conventional methods for BSEEG calculation required the use of analytical software, which limited their practical application to research facilities. To facilitate the dissemination of this BSEEG method to support clinical practice, we have developed and launched a web-based BSEEG analysis system (https://bseeg.shinozakilab.net/). This platform is designed to facilitate the collection and analysis of BSEEG data in busy clinical settings. By enabling healthcare professionals, including physicians and nurses, to upload EEG data via a web browser and display BSEEG scores at the bedside, this system could significantly improve accessibility to the BSEEG method in clinical practice [72].

## EEG in a Rodent Model of Delirium

One of the challenges hampering the advancement of research in the field of delirium is the lack of robust animal models for investigating its pathophysiology or to test potential interventions. From this standpoint, using EEG to assess and quantify delirium-like states in animals can be a breakthrough. There have been several early studies trying such approaches, with more recent advancements include the application of the BSEEG method to mouse models. Trzepacz et al. conducted a study in which they administered atropine to rats and performed behavioral observations and EEG measurements [73]. The study confirmed changes in behavior, such as attention deficit, disrupted sleep-wake rhythms, and aimless movements, as well as alterations in activity levels. EEG revealed the appearance of slow waves and high-amplitude irregular waves. These findings suggested that atropine administration could induce a delirium-like state, establishing it as an animal model for delirium research. Additionally, Leavitt et al. demonstrated that this atropine-induced delirium model in rats exhibited a dose-dependent increase in EEG slow wave components and cognitive impairment [74]. Tamura et al. reported an increase in slow-wave components in rats in rats under delirium induced by biperiden, suggesting that EEG may be useful for detecting delirium in animals [75]. Semmler et al. studied an LPS-induced delirium model that encompassed slowing of mental processes, impaired attention, disorientation, delirium, and coma [76]. The study demonstrated a decrease in overall spectral frequency accompanied by suppression of alpha waves and increased delta power.

Based on these studies, EEG has gained attention in delirium research. Cunningham et al. studied detailed spatial and temporal electrophysiological features in the LPS-induced delirium model rats using local field potential measurements [77]. Banks et al. studied the effects of inflammation associated with postoperative delirium by administering LPS to mice. They reproduced the association between inflammation and cortical slow wave activity in a mouse model [78]. In such studies investigating how inflammation and neural activity are associated with delirium, EEG has emerged as an important indicator of delirium, and with significant potential for elucidating delirium pathophysiology.

Following the successful development of BSEEG method in human delirium studies [63,66], we conducted a study to adapt the BSEEG method to animal models. First, we focused on the LPS-induced mouse models as a model that simulates delirium in infections and sepsis. In our study, regular diurnal fluctuations in BSEEG scores consistent with the circadian rhythm were observed in mice at baseline, and LPS administration increased BSEEG scores and disrupted the circadian rhythm. Additionally, the increase in BSEEG scores tended to be greater in aged mice than in young ones [79]. Next, we conducted a study using the LPS-induced delirium model to evaluate the correlation between BSEEG and delirium-like behaviors as well as microglial activity in brain tissue. Behavioral tests revealed attention deficits and reduced activity levels in young mice, with a significant correlation observed between these behaviors and BSEEG scores. In contrast, aged mice exhibited marked reductions in activity levels, and most behavioral tests were not feasible. However, an increase in BSEEG scores was observed. This suggests that the BSEEG method may be able to detect delirium even when behavioral tests are difficult to evaluate due to low activity. Additionally, microglia in brain tissue were activated by LPS administration, and this activation correlated with BSEEG scores [80]. Following successful validation of the BSEEG method in a mouse model of deliriums, we evaluated the effects of potential preventive candidates, P7C3-A20 and minocycline, using the BSEEG score in the LPS-induced delirium model. In aged mice, these compounds both suppressed of the LPS-induced increase in BSEEG scores [81]. This study demonstrated the usefulness of the BSEEG score as an indicator for intervention response. Furthermore, we evaluated the BSEEG score after EEG-head-mount implantation surgery as a postoperative delirium model. The average BSEEG scores were higher at most postoperative time points in aged mice than in young mice, and aged mice required longer to return to a steady state [82]. Additionally, we evaluated the relationship between BSEEG scores and delirium-like behavior in this postoperative delirium model. In young mice, attention deficits and reduced activity levels were observed, and a correlation with BSEEG scores was significant. In contrast, in aged mice, although BSEEG scores were elevated, reduced activity levels were prominent, similar to the case of LPS induced model. These results suggest that the BSEEG score reflects the ability to accurately measure physiological changes in the brain, even when motor activity is impaired and accurate behavioral assessment is hindered [83].

It is worth noting that several well-designed studies have reported the usefulness of EEG in animal experiments for detecting delirium in recent years. Harrison et al. used a polymicrobial cecal slurry method as a sepsis model in mice. They observed changes in EEG during sleep and wakefulness and in behavioral tests. Furthermore, impairments in hippocampal long-term potentiation were also observed [84]. Lunardi et al. demonstrated that simulated surgery, anesthesia, and intensive care environments induced sleep fragmentation, EEG slowing, and circadian rhythm disruption in aged mice using EEG. Furthermore, they observed reduced expression of circadian rhythm-related genes [85]. Kimchi et al. conducted a neurophysiological study using rats to examine delirium models induced by scopolamine and LPS administration. The rats exhibited EEG slowing as well as impaired attention and cognitive function, with increased motor activity in the scopolamine group and decreased motor activity in the LPS group [86].

As shown above, assessing “delirium” in model animals with EEG, rather than behavioral tests, captures the pathophysiology of delirium more accurately. Thus, such EEG based approaches are vital for elucidating its mechanisms and developing novel therapeutics. Once promising interventions are identified in animal models using EEG quantification, subsequent clinical trials with the same EEG-based assessments will enable direct translation, allowing objective quantification of treatment response and ultimately leading to effective delirium therapies.

## Future horizons

The assessment of delirium using electroencephalography (EEG) provides an objective measure of the brain's electrical activity, enabling more accurate detection of delirium in patients. Furthermore, it may allow for the detection of potential indicators of poor prognosis in delirium patients with higher sensitivity than clinical symptom-based diagnosis. Using compact EEG devices equipped with a limited number of electrodes enables short-term and low-effort usage by minimally trained medical staff, making it possible to apply them widely and repeatedly to large numbers of patients at high-risk of delirium both inside and outside hospitals. POC-EEG could be utilized as the “sixth vital sign,” following heart rate, blood pressure, body temperature, respiratory rate, and pain. This would enable early detection and intervention for delirium, ultimately improving patient outcomes. Such an approach could lead to better survival, shortened hospital stays and reduced medical costs, thereby significantly improving the healthcare economy.

To bring this technology to patient bedsides, it is crucial to make the approach user-friendly so frontline healthcare staff are willing to adopt it. For that goal, smaller and lighter devices with shorter recording times are under development. Single channel EEG signal has been proven sufficient to capture delirium and less than 1 ​min recording with advanced signal processing has become feasible. Smartphone applications could be developed as demonstrated above, bringing this goal closer to realization.

At the same time, it is worth noting that one of major challenges of POC-EEG lies in its accuracy. For instance, based on our BSEEG data, there can be a large overlap between delirium and non-delirium states, sometimes making it difficult to establish an ideal cutoff value. This issue should be addressed by improving the technology of EEG devices, enhancing electrode quality, and refining the algorithms used to quantify EEG data. Moreover, since the baseline states may vary significantly among individuals, longitudinal data collection will be necessary to obtain baseline scores for each individual and establish population norms, similar to the case of blood pressure. In order to repeatedly measure EEG data from a large number of samples, the simplicity of the data acquisition method as mentioned above remains critical. Therefore, further development of POC-EEG must be pursued to advance this field.

## Conclusion

Bedside EEG technologies are poised to transform future clinical practice. Rigorous comparative trials incorporating robust animal-to-human translational pipelines will determine which platform(s) will become standard-of-care for delirium diagnosis and therapeutic monitoring in the near future.

## Author contribution

Takehiko Yamanashi drafted the initial draft, finalized the document and figures. Tsuyoshi Nishiguchi drafted the initial draft. Gen Shinozaki drafted the initial draft, finalized the document and figures.

## Declaration of generative AI and AI-assisted technologies in the writing process

During the preparation of this work, the authors used ChatGPT (GPT-5, OpenAI, San Francisco, CA, USA) in order to improve the readability of the content and avoid grammatical errors. In addition, Fig. 1 was created with the assistance of ChatGPT5. After using this tool/service, the authors reviewed and edited the content as needed and take full responsibility for the content of the publication.

## Declaration of competing interest

The authors declare the following financial interests/personal relationships which may be considered as potential competing interests: Gen Shinozaki reports financial support was provided by NIH. Gen Shinozaki reports financial support was provided by Sumitomo Pharma Co Ltd. Gen Shinozaki reports a relationship with Delight Health Inc. that includes: equity or stocks. Gen Shinozaki reports a relationship with Predelix Medical LLC that includes: equity or stocks. Gen Shinozaki has patent Non-invasive device for predicting and screening delirium pending to University of Iowa Research Foundation. Gen Shinozaki has patent Prediction of patient outcomes with a novel electroencephalography device pending to N/A. Gen Shinozaki has patent DEVICES, SYSTEMS, AND METHOD FOR QUANTIFYING NEURO-INFLAMMATION pending to N/A. If there are other authors, they declare that they have no known competing financial interests or personal relationships that could have appeared to influence the work reported in this paper.

## References

[1] Inouye S.K. (2006). Delirium in older persons. N Engl J Med.

[2] Pisani M.A., McNicoll L., Inouye S.K. (2003). Cognitive impairment in the intensive care unit. Clin Chest Med.

[3] Boustani M., Baker M.S., Campbell N., Munger S., Hui S.L., Castelluccio P. (2010). Impact and recognition of cognitive impairment among hospitalized elders. J Hosp Med.

[4] McCusker J., Cole M.G., Dendukuri N., Belzile E. (2003). Does delirium increase hospital stay?. J Am Geriatr Soc.

[5] Yamanashi T., Iwata M., Crutchley K.J., Sullivan E.J., Malicoat J.R., Anderson Z.M. (2021). New cutoff scores for delirium screening tools to predict patient mortality. J Am Geriatr Soc.

[6] Inouye S.K., Westendorp R.G., Saczynski J.S. (2014). Delirium in elderly people. Lancet (London, England).

[7] McCusker J., Cole M., Abrahamowicz M., Primeau F., Belzile E. (2002). Delirium predicts 12-month mortality. Arch Intern Med.

[8] Fong T.G., Tulebaev S.R., Inouye S.K. (2009). Delirium in elderly adults: diagnosis, prevention and treatment. Nat Rev Neurol.

[9] American Psychiatric A (2022). Text Revision.

[10] Mansutti I., Muzzana C., Vater V., Dettwiler P.U., Palese A., Ausserhofer D. (2025 Jun 28). Delirium in nursing homes and long-term care facilities: findings of a scoping review of detection tools. Eur Geriatr Med.

[11] Kim S., Choi E., Jung Y., Jang I. (2023). Postoperative delirium screening tools for post-anaesthetic adult patients in non-intensive care units: a systematic review and meta-analysis. J Clin Nurs.

[12] Lin C.J., Su I.C., Huang S.W., Chen P.Y., Traynor V., Chang H.R. (2023). Delirium assessment tools among hospitalized older adults: a systematic review and metaanalysis of diagnostic accuracy. Ageing Res Rev.

[13] Inouye S.K., van Dyck C.H., Alessi C.A., Balkin S., Siegal A.P., Horwitz R.I. (1990). Clarifying confusion: the confusion assessment method. A new method for detection of delirium. Ann Intern Med.

[14] Ely E.W., Inouye S.K., Bernard G.R., Gordon S., Francis J., May L. (2001). Delirium in mechanically ventilated patients: validity and reliability of the confusion assessment method for the intensive care unit (CAM-ICU). JAMA.

[15] Marcantonio E.R., Ngo L.H., O'Connor M., Jones R.N., Crane P.K., Metzger E.D. (2014). 3D-CAM: derivation and validation of a 3-minute diagnostic interview for CAM-defined delirium: a cross-sectional diagnostic test study. Ann Intern Med.

[16] Fick D.M., Inouye S.K., Guess J., Ngo L.H., Jones R.N., Saczynski J.S. (2015). Preliminary development of an ultrabrief two-item bedside test for delirium. J Hosp Med.

[17] Bellelli G., Morandi A., Davis D.H., Mazzola P., Turco R., Gentile S. (2014). Validation of the 4AT, a new instrument for rapid delirium screening: a study in 234 hospitalised older people. Age Ageing.

[18] Gaudreau J.D., Gagnon P., Harel F., Tremblay A., Roy M.A. (2005). Fast, systematic, and continuous delirium assessment in hospitalized patients: the nursing delirium screening scale. J Pain Symptom Manag.

[19] Schuurmans M.J., Shortridge-Baggett L.M., Duursma S.A. (2003). The Delirium observation screening scale: a screening instrument for delirium. Res Theor Nurs Pract.

[20] Sands M.B., Dantoc B.P., Hartshorn A., Ryan C.J., Lujic S. (2010). Single Question in Delirium (SQiD): testing its efficacy against psychiatrist interview, the confusion assessment method and the memorial delirium assessment scale. Palliat Med.

[21] Trzepacz P.T., Mittal D., Torres R., Kanary K., Norton J., Jimerson N. (2001). Validation of the delirium rating Scale-revised-98: comparison with the delirium rating scale and the cognitive test for delirium. J Neuropsychiatry Clin Neurosci.

[22] Breitbart W., Rosenfeld B., Roth A., Smith M.J., Cohen K., Passik S. (1997). The memorial delirium assessment scale. J Pain Symptom Manag.

[23] Smith C.J., Hodge D., Harrison F.E., Williams Roberson S. (2024). The pathophysiology and biomarkers of delirium. Semin Neurol.

[24] Engel G.L., Romano J. (1944). DELIRIUM: II. Reversibility of the electroencephalogram with experimental procedures. Arch Neurol Psychiatry (Chic).

[25] Romano J., Engel G.L. (1944). DELIRIUM: I. Electroencephalographic data. Arch Neurol Psychiatry (Chic).

[26] Engel G.L., Romano J. (1959). Delirium, a syndrome of cerebral insufficiency. J Chron Dis.

[27] Boord M.S., Moezzi B., Davis D., Ross T.J., Coussens S., Psaltis P.J. (2021). Investigating how electroencephalogram measures associate with delirium: a systematic review. Clin Neurophysiol : Off J Int Feder Clin Neurophys.

[28] Wiegand T.L.T., Rémi J., Dimitriadis K. (2022). Electroencephalography in delirium assessment: a scoping review. BMC Neurol.

[29] Koponen H., Partanen J., Pääkkönen A., Mattila E., Riekkinen P.J. (1989). EEG spectral analysis in delirium. J Neurol Neurosurg Psychiatry.

[30] Jacobson S.A., Leuchter A.F., Walter D.O. (1993). Conventional and quantitative EEG in the diagnosis of delirium among the elderly. J Neurol Neurosurg Psychiatry.

[31] Sutter R., Kaplan P.W. (2013). Clinical and electroencephalographic correlates of acute encephalopathy. J Clin Neurophysiol : Off Pub Am Electroencephal Soci.

[32] van der Kooi A.W., Zaal I.J., Klijn F.A., Koek H.L., Meijer R.C., Leijten F.S. (2015). Delirium detection using EEG: what and how to measure. Chest.

[33] Fleischmann R., Tränkner S., Bathe-Peters R., Rönnefarth M., Schmidt S., Schreiber S.J. (2019). Diagnostic performance and utility of quantitative EEG analyses in delirium: confirmatory results from a large retrospective case-control study. Clin EEG Neurosci.

[34] Hunter A., Crouch B., Webster N., Platt B. (2020). Delirium screening in the intensive care unit using emerging QEEG techniques: a pilot study. AIMS Neurosci.

[35] Keijzer H.M., Klop M., van Putten M., Hofmeijer J. (2020). Delirium after cardiac arrest: phenotype, prediction, and outcome. Resuscitation.

[36] Numan T., Slooter A.J.C., van der Kooi A.W., Hoekman A.M.L., Suyker W.J.L., Stam C.J. (2017). Functional connectivity and network analysis during hypoactive delirium and recovery from anesthesia. Clin Neurophysiol : Off J Int Feder Clin Neurophys.

[37] Plaschke K., Hill H., Engelhardt R., Thomas C., von Haken R., Scholz M. (2007). EEG changes and serum anticholinergic activity measured in patients with delirium in the intensive care unit. Anaesthesia.

[38] Reischies F.M., Neuhaus A.H., Hansen M.L., Mientus S., Mulert C., Gallinat J. (2005). Electrophysiological and neuropsychological analysis of a delirious state: the role of the anterior cingulate gyrus. Psychiatry Res.

[39] Tanabe S., Mohanty R., Lindroth H., Casey C., Ballweg T., Farahbakhsh Z. (2020). Cohort study into the neural correlates of postoperative delirium: the role of connectivity and slow-wave activity. Br J Anaesth.

[40] Thomas C., Hestermann U., Kopitz J., Plaschke K., Oster P., Driessen M. (2008). Serum anticholinergic activity and cerebral cholinergic dysfunction: an EEG study in frail elderly with and without delirium. BMC Neurosci.

[41] Thomas C., Hestermann U., Walther S., Pfueller U., Hack M., Oster P. (2008). Prolonged activation EEG differentiates dementia with and without delirium in frail elderly patients. J Neurol Neurosurg Psychiatry.

[42] Fleischmann R., Traenkner S., Kraft A., Schmidt S., Schreiber S.J., Brandt S.A. (2019). Delirium is associated with frequency band specific dysconnectivity in intrinsic connectivity networks: preliminary evidence from a large retrospective pilot case-control study. Pilot Feas Studies.

[43] Nielsen R.M., Urdanibia-Centelles O., Vedel-Larsen E., Thomsen K.J., Møller K., Olsen K.S. (2020). Continuous EEG monitoring in a consecutive patient cohort with sepsis and delirium. Neurocritical Care.

[44] Carrarini C., Calisi D., De Rosa M.A., Di Iorio A., D'Ardes D., Pellegrino R. (2023). QEEG abnormalities in cognitively unimpaired patients with delirium. J Neurol Neurosurg Psychiatry.

[45] Guay C.S., Kafashan M., Huels E.R., Jiang Y., Beyoglu B., Spencer J.W. (2023). Postoperative delirium severity and recovery correlate with electroencephalogram spectral features. Anesth Analg.

[46] Kimchi E.Y., Neelagiri A., Whitt W., Sagi A.R., Ryan S.L., Gadbois G. (2019). Clinical EEG slowing correlates with delirium severity and predicts poor clinical outcomes. Neurology.

[47] van Sleuwen M., Sun H., Eckhardt C., Neelagiri A., Tesh R.A., Westmeijer M. (2022). Physiological assessment of delirium severity: the electroencephalographic confusion assessment method severity score (E-CAM-S). Crit Care Med.

[48] Tesh R.A., Sun H., Jing J., Westmeijer M., Neelagiri A., Rajan S. (2022). VE-CAM-S: visual EEG-based grading of delirium severity and associations with clinical outcomes. Crit Care Exp.

[49] Fratangelo R., Lolli F., Scarpino M., Grippo A. (2025). Point-of-Care electroencephalography in acute neurological care: a narrative review. Neurol Int.

[50] Katz I.R., Mossey J., Sussman N., Muenz L., Harner R., Curlik S. (1991). Bedside clinical and electrophysiological assessment: assessment of change in vulnerable patients. Int Psychogeriatr.

[51] Matsushima E., Nakajima K., Moriya H., Matsuura M., Motomiya T., Kojima T. (1997). A psychophysiological study of the development of delirium in coronary care units. Biol Psychiatry.

[52] Ely E.W., Truman B., Manzi D.J., Sigl J.C., Shintani A., Bernard G.R. (2004). Consciousness monitoring in ventilated patients: bispectral EEG monitors arousal not delirium. Intensive Care Med.

[53] Plaschke K., Fichtenkamm P., Schramm C., Hauth S., Martin E., Verch M. (2010). Early postoperative delirium after open-heart cardiac surgery is associated with decreased bispectral EEG and increased cortisol and interleukin-6. Intensive Care Med.

[54] Bao L., Liu T., Zhang Z., Pan Q., Wang L., Fan G. (2023). The prediction of postoperative delirium with the preoperative bispectral index in older aged patients: a cohort study. Aging Clin Exp Res.

[55] Luo A., Muraida S., Pinchotti D., Richardson E., Ye E., Hollingsworth B. (2021). Bispectral index monitoring with density spectral array for delirium detection. J Acad Consul-Liaison Psych.

[56] de Burlo R., Robles A., Salazar S., Yang J., MacDonald S., Luo A. (2025 Jul-Aug). Validation of cerebral state monitor frequency power ratios for detection of delirium. J Acad Consult Liaison Psychiatry.

[57] Numan T., van den Boogaard M., Kamper A.M., Rood P.J.T., Peelen L.M., Slooter A.J.C. (2019). Delirium detection using relative Delta power based on 1-minute single-channel EEG: a multicentre study. Br J Anaesth.

[58] Ditzel F.L., Hut S.C.A., van den Boogaard M., Boonstra M., Leijten F.S.S., Wils E.J. (2024). DeltaScan for the assessment of acute encephalopathy and delirium in ICU and non-ICU patients, a prospective cross-sectional multicenter validation study. Am J Geriatr Psychiatr : Offi J Am Ass Geriat Psych.

[59] Mulkey M.A., Gantt L.T., Hardin S.R., Munro C.L., Everhart D.E., Kim S. (2022). Rapid handheld continuous electroencephalogram (EEG) has the potential to detect delirium in older adults. Dimens Crit Care Nurs : DCCN.

[60] Mulkey M., Albanese T., Kim S., Huang H., Yang B. (2022). Delirium detection using GAMMA wave and machine learning: a pilot study. Res Nurs Health.

[61] Mulkey M.A., Huang H., Albanese T., Kim S., Yang B. (2023). Supervised deep learning with vision transformer predicts delirium using limited lead EEG. Sci Rep.

[62] Wijnen V.J.M., Oudewortel L., van Luijtelaar G., Witlox J., Slooter A.J.C., van Gool W.A. (2022). Feasibility and potential of a bedside mini-EEG for diagnosing delirium superimposed on dementia. Clin Neurophysiol : Off J Int Feder Clin Neurophys.

[63] Shinozaki G., Chan A.C., Sparr N.A., Zarei K., Gaul L.N., Heinzman J.T. (2018). Delirium detection by a novel bispectral electroencephalography device in general hospital. Psychiatr Clin Neurosci.

[64] Lee S., Yuki K., Chan A., Cromwell J., Shinozaki G. (2019). The point-of-care EEG for delirium detection in the emergency department. Am J Emerg Med.

[65] Zarei K., Sparr N.A., Trapp N.T., Neuhaus E.D., Cromwell J.W., Boes A.D. (2020). Bispectral EEG (BSEEG) to assess arousal after electro-convulsive therapy (ECT). Psychiatry Res.

[66] Shinozaki G., Bormann N.L., Chan A.C., Zarei K., Sparr N.A., Klisares M.J. (2019). Identification of patients with high mortality risk and prediction of outcomes in delirium by bispectral EEG. J Clin Psychiatr.

[67] Yamanashi T., Crutchley K.J., Wahba N.E., Sullivan E.J., Comp K.R., Kajitani M. (2022). Evaluation of point-of-care thumb-size bispectral electroencephalography device to quantify delirium severity and predict mortality. Br J Psychiatr.

[68] Nishizawa Y., Yamanashi T., Saito T., Marra P., Crutchley K.J., Wahba N.E. (2023). Bispectral EEG (BSEEG) algorithm captures high mortality risk among 1,077 patients: its relationship to delirium motor subtype. Am J Geriatr Psychiatr : Offi J Am Ass Geriat Psych.

[69] Yamanashi T., Kajitani M., Iwata M., Crutchley K.J., Marra P., Malicoat J.R. (2021). Topological data analysis (TDA) enhances bispectral EEG (BSEEG) algorithm for detection of delirium. Sci Rep.

[70] Saito T., Malicoat J.R., Leyden L.R., Williams J.C., Jellison S.S., Long H. (2021). Mortality prediction by bispectral electroencephalography among 502 patients: its role in dementia. Brain Commun.

[71] Yamanashi T., Marra P.S., Crutchley K.J., Wahba N.E., Malicoat J.R., Sullivan E.J. (2021). Mortality among patients with sepsis associated with a bispectral electroencephalography (BSEEG) score. Sci Rep.

[72] Shimura A., Seki T., Nishiguchi T., Yamanishi K., Ishii T., Aoyama B. (2024). A web-based delirium detection application using bispectral electroencephalography (BSEEG). Gen Hosp Psychiatry.

[73] Trzepacz P.T., Leavitt M., Ciongoli K. (1992). An animal model for delirium. Psychosomatics.

[74] Leavitt M.L., Trzepacz P.T., Ciongoli K. (1994). Rat model of delirium: atropine dose-response relationships. J Neuropsychiatry Clin Neurosci.

[75] Tamura Y., Chiba S., Takasaki H., Tabata K., Ishimaru Y., Ishimoto T. (2006). Biperiden-induced delirium model in rats: a behavioral and electroencephalographic study. Brain Res.

[76] Semmler A., Hermann S., Mormann F., Weberpals M., Paxian S.A., Okulla T. (2008). Sepsis causes neuroinflammation and concomitant decrease of cerebral metabolism. J Neuroinflammation.

[77] Mamad O., Islam M.N., Cunningham C., Tsanov M. (2018). Differential response of hippocampal and prefrontal oscillations to systemic LPS application. Brain Res.

[78] Sultan Z.W., Jaeckel E.R., Krause B.M., Grady S.M., Murphy C.A., Sanders R.D. (2021). Electrophysiological signatures of acute systemic lipopolysaccharide-induced inflammation: potential implications for delirium science. Br J Anaesth.

[79] Yamanashi T., Malicoat J.R., Steffen K.T., Zarei K., Li R., Purnell B.S. (2021). Bispectral EEG (BSEEG) quantifying neuro-inflammation in mice induced by systemic inflammation: a potential mouse model of delirium. J Psychiatr Res.

[80] Nishiguchi T., Yamanishi K., Gorantla N., Shimura A., Seki T., Ishii T. (2024). Lipopolysaccharide-induced delirium-like behavior and microglial activation in mice correlate with bispectral electroencephalography. J Ger Series A, Biolog Sci Med Sci.

[81] Nishiguchi T., Yamanishi K., Patel S., Malicoat J.R., Phuong N.J., Seki T. (2024). Discovery of novel protective agents for infection-related delirium through bispectral electroencephalography. Transl Psychiatry.

[82] Nishiguchi T., Shibata K., Yamanishi K., Dittrich M.N., Islam N.Y., Patel S. (2024). The bispectral electroencephalography method quantifies postoperative delirium-like states in young and aged Male mice after head-mount implantation surgery. J Gerontol A Biol Sci Med Sci.

[83] Nishiguchi T., Yamanishi K., Shimura A., Seki T., Ishii T., Aoyama B. (2025). The age-related susceptibility to postoperative delirium quantified by bispectral electroencephalography correlates with postoperative delirium-like behavior in mice. Psych Clin Neurosci Reports.

[84] Consoli D.C., Spitznagel B.D., Owen B.M., Kang H., Williams Roberson S., Pandharipande P. (2022). Altered EEG, disrupted hippocampal long-term potentiation and neurobehavioral deficits implicate a delirium-like state in a mouse model of sepsis. Brain Behav Immun.

[85] Dulko E., Jedrusiak M., Osuru H.P., Atluri N., Illendula M., Davis E.M. (2023). Sleep fragmentation, electroencephalographic slowing, and circadian disarray in a mouse model for intensive care unit delirium. Anesth Analg.

[86] Kimchi E., Coughlin B., Cash S. (2017). Rodent models of delirium and encephalopathy: behavioral and neurophysiological studies in aging (P5. 090). Neurology.

